# A generic method for improving the spatial interoperability of medical and ecological databases

**DOI:** 10.1186/s12942-017-0109-5

**Published:** 2017-10-03

**Authors:** A. Ghenassia, J. B. Beuscart, G. Ficheur, F. Occelli, E. Babykina, E. Chazard, M. Genin

**Affiliations:** 1 EA 2694 - Santé publique : épidémiologie et qualité des soins, University of Lille, 59000 Lille, France; 2 EA 4483 - Impact de l’environnement chimique sur la santé humaine, University of Lille, 59000 Lille, France; 3 Department of Public Health, CHU Lille, 59000 Lille, France

**Keywords:** Spatial analysis, Data reuse, Change-of-support problem, Interoperability

## Abstract

**Background:**

The availability of big data in healthcare and the intensive development of data reuse and georeferencing have opened up perspectives for health spatial analysis. However, fine-scale spatial studies of ecological and medical databases are limited by the change of support problem and thus a lack of spatial unit interoperability. The use of spatial disaggregation methods to solve this problem introduces errors into the spatial estimations. Here, we present a generic, two-step method for merging medical and ecological databases that avoids the use of spatial disaggregation methods, while maximizing the spatial resolution.

**Methods:**

Firstly, a mapping table is created after one or more transition matrices have been defined. The latter link the spatial units of the original databases to the spatial units of the final database. Secondly, the mapping table is validated by (1) comparing the covariates contained in the two original databases, and (2) checking the spatial validity with a spatial continuity criterion and a spatial resolution index.

**Results:**

We used our novel method to merge a medical database (the French national diagnosis-related group database, containing 5644 spatial units) with an ecological database (produced by the French National Institute of Statistics and Economic Studies, and containing with 36,594 spatial units). The mapping table yielded 5632 final spatial units. The mapping table’s validity was evaluated by comparing the number of births in the medical database and the ecological databases in each final spatial unit. The median [interquartile range] relative difference was 2.3% [0; 5.7]. The spatial continuity criterion was low (2.4%), and the spatial resolution index was greater than for most French administrative areas.

**Conclusions:**

Our innovative approach improves interoperability between medical and ecological databases and facilitates fine-scale spatial analyses. We have shown that disaggregation models and large aggregation techniques are not necessarily the best ways to tackle the change of support problem.

**Electronic supplementary material:**

The online version of this article (doi:10.1186/s12942-017-0109-5) contains supplementary material, which is available to authorized users.

## Background

In the field of epidemiology, the term “spatial analysis” refers to the description and analysis of the spatial distribution of healthcare phenomena, such as the incidence or prevalence of disease or healthcare consumption across geographic areas [1–5]. Although spatial analysis can be applied to point data, geostatistical data and aggregated data, most of the data for spatial analysis in the field of health are aggregated because they ensure that the patients’ data remain confidential. By definition, these so-called ecological studies use data that have been aggregated into administrative spatial units, such as counties, provinces and states. These analyses require two categories of aggregated data. The first category is related to how the events (e.g. the cases of disease or surgical acts) are counted within each spatial unit in the study area. The second category is related to the descriptive ecological data on the source population and the living environment within these spatial units, such as the socio-economic level, the employment rate, housing conditions and environmental quality. For example, a spatial analysis of the incidence of Crohn’s disease in northern France examined correlations between two data sources: all new cases of Crohn’s disease recorded in the EPIMAD register for each district (*canton*), and the characteristics of each of these districts in terms of the underlying population and the living environment. By combining these two sources, the investigators were able to (1) calculate the incidence of Crohn’s disease for each *canton*, and (2) evaluate the influence of the living environment and the population’s socio-economic level [6, 7].

Spatial analysis in healthcare is attracting growing interest because of improvements in statistical analysis, the development of information technology tools, and the emergence of disease registries [8–14]. More recently, the availability of big data in healthcare [15–17] and the intensive development of data reuse [18, 19] and georeferencing [20, 21] have opened up new perspectives for describing healthcare consumption or disease prevalence/incidence over large geographical areas—even whole countries—and analyzing their ecological determinants (such as socio-economic factors) [22, 23].

However, the correlation of big data and ecological data over large areas is complicated by the problem of database interoperability [24–26]. In the specific setting of spatial analysis, interoperability is based on the smallest possible spatial reference unit, which acts as a link between the medical database and the ecological database. In the absence of this link, the data must be aggregated on a larger scale, which limits the precision of the results [27–29]. In fact, the quality and relevance of the conclusions of a spatial analysis depend on the concordance between the spatial resolution and the nature of the phenomenon studied. The use of aggregated data induces an ecological bias that fades (but does not disappear) when the spatial resolution is increased [30]. Moreover, a finer-scale analysis enables the assessment of more local phenomena, such as the impact of sources of pollution [31]. However, larger spatial units may be more appropriate if the underlying disease pathways involve larger-scale phenomena. The availability of fine-scale data provides an opportunity to use the scale that best matches the study’s goal.

Poor interoperability between medical databases and ecological databases thus appears to be a major limitation for fine-scale spatial analyses of large geographical areas. However, the interoperability problem should not limit the choice of the most appropriate scale. This interoperability problem has been highlighted (for example) for National Health Service data in the UK, Statewide Planning and Research Cooperative System data from New York State in the USA, and the French national diagnosis-related group database (*Programme Médicalisé des Systèmes d’Information*, PMSI) [27, 32, 33].

Two ways of tackling the interoperability problem have been suggested: spatial disaggregation and spatial aggregation. The first approach consists in creating a mapping table that adopts the finest scale; consequently, the data aggregated on a larger scale are disaggregated into spatial units at the finest scale. However, this necessitates the use of complex statistical models for spatial disaggregation (such as areal interpolation models) to estimate the variables’ values on a smaller scale. Hence, these procedures can lead to errors in the spatial estimation, which are especially large because the spatial units of origin are considered on very different scales (e.g. by going from the state scale to the town scale) [26, 34]. The second approach (aggregation methods) consists in creating a mapping table that links the spatial units of one or both databases to a larger scale. In a simple, particular case, the data from one of the two databases are aggregated to the spatial scale of the other database. However, in the most frequent case, the spatial units of the two databases are aggregated into a larger spatial unit that covers them both. Although most studies use administrative spatial units as a larger spatial unit, this is not necessarily the finest and/or most appropriate scale for use. Consequently, aggregation methods markedly decrease spatial resolution (e.g. by going from the town scale to the county scale), and may lead to an increase in the ecological bias [27–29].

The primary objective of the present study was to develop and characterize a generic method for building a mapping table between a medical database and an ecological database while maximizing the spatial resolution and avoiding the use of spatial disaggregation techniques and thus enabling the choice of most appropriate scale for the phenomenon being studied. By way of an illustrative example, we applied this method to the interoperability of the above-mentioned PMSI medical database and the socio-economic data produced by the French National Institute of Statistics and Economic Studies (*Institut National de la Statistique et des Études Économiques*, INSEE).

## The generic method

**Fig. 1:**
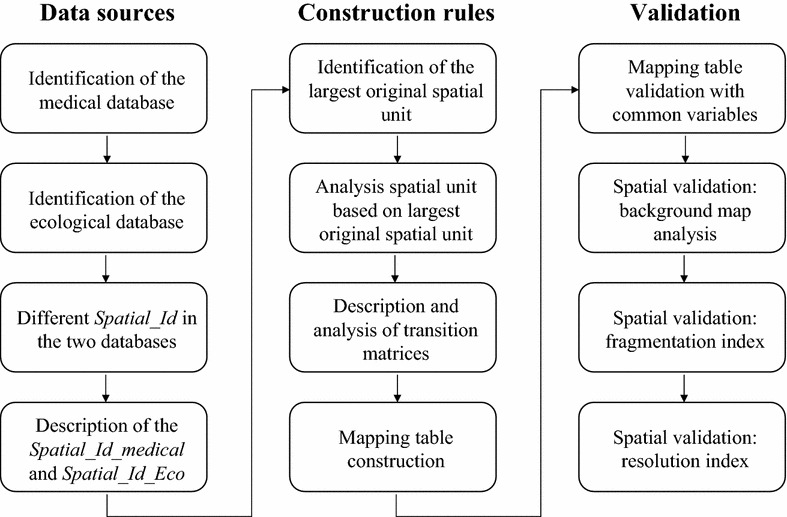
A standardized approach for maximizing the interoperability of ecological and medical databases for spatial analysis

This section describes the generic method for improving the spatial interoperability of medical and ecological databases. The different steps in this generic method are summarized in Fig. 1.

### Data and objectives

Let us consider two distinct databases: a medical database that describes patients and healthcare events, and an ecological database that describes the population. The present method considers the following conditions of application:

1. The medical database is organized on the scale of the individual. Each individual is attached to a spatial ID *Spatial_Id_Medical*, which corresponds to the spatial unit *SU_medical*. A variable characterizes each healthcare event.
2. The ecological database is organized on the scale of the spatial unit *SU_eco,* which has a unique spatial ID *Spatial_Id_Eco*.
3. The spatial units *SU_medical* and *SU_eco* differ, as do the spatial IDs *Spatial_Id_Eco* and *Spatial_Id_Medical*.

**Fig. 2:**
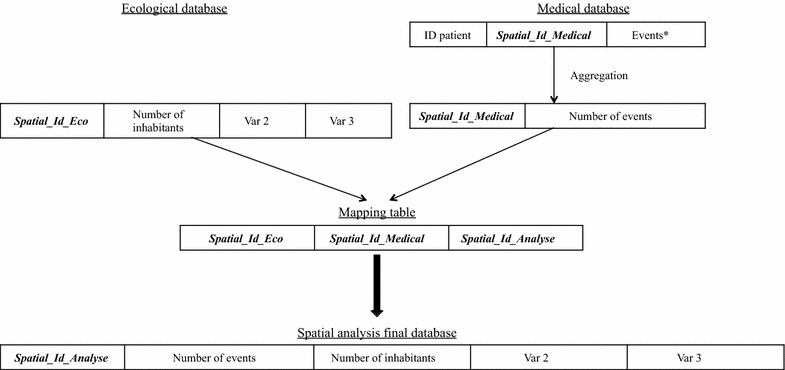
A generic method for building a final database for spatial analysis. Asterisk a variable characterizing the healthcare event studied (e.g. cases of disease, length of hospital stay, surgical acts, etc.)

The objective of our method is to build a mapping table that enables the creation of a final database comprising both medical and ecological data from the above-mentioned databases on the scale of the spatial unit *SU_analysis* and with a unique spatial ID called *Spatial_Id_Analysis*. The medical database must be aggregated for the variable characterizing the healthcare event on the scale of the spatial unit *SU_medical* (Fig. 2). An example showing how the final spatial analysis database is built is provided in the Additional file 1.

### Construction rules

**Fig. 3:**
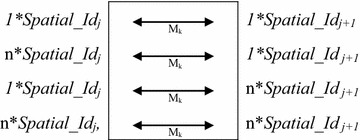
Mutually exclusive equivalence situations for a transition matrix M_k_. M_k_ is a transition matrix from among M_1_…M_p_ transition matrices. M_k_ describes the relationship between a *Spatial_Id*_*j*_ and a *Spatial_Id*_*j*+1_ and therefore links them. n > 1, corresponds to the number of spatial IDs

1. *The direction of the relationship*. When spatial units differ in size (i.e. *SU_medical* ≠ *SU_eco*), the two databases can only be aligned after the data have been aggregated. Count data are aggregated by calculating a sum, whereas continuous variables or proportions can be aggregated by calculating a median, mean or weighted mean. The larger of the two spatial units is then chosen as *SU_analysis*. The reverse process requires the use of a disaggregation method, leading to a loss of precision [34, 35].
2. *Transition matrices M*_1_ *…M*_*p*_. A transition matrix is a tool for linking an original spatial ID to a final spatial ID:
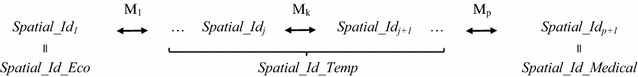

A mapping table for the IDs *Spatial_Id_Medical* and *Spatial_Id_Eco* IDs can be built by using *p* transition matrices (*p* ≥ 1). For example, a transition matrix makes it possible to associate each town’s spatial ID with the spatial ID of the state to which it belongs. However, in more complex situations, there may be no direct way of linking the two spatial IDs. Thus, two or more matrices are required, leading to the creation of at least one temporary spatial ID *Spatial_Id_Temp*. The mapping table yields *p* + 1 *Spatial_Id,* where *Spatial_Id*_1_ corresponds to the *Spatial_Id_Eco* and *Spatial_Id*_*p*+1_ corresponds to the *Spatial_Id_Medical*. The transition matrices are based on a detailed assessment of the *Spatial_Id_Medical* and *Spatial_Id_Eco* IDs. It is then necessary to describe all the equivalence situations for each transition matrix. One or several *Spatial_Id*_*j*_ can correspond to one or several *Spatial_Id*_*j*+1_ (1 ≤ j < p + 1). The various, mutually exclusive equivalence situations for a given transition matrix M_k_ (1 ≤ k ≤ *p*) are shown in Fig. 3.

### Validation

#### Validation of the mapping table

After the final database has been built, it is necessary to validate the quality of the interface between the medical database and the ecological database. We used the following approach: (1) identification of the set of variables shared by the medical database and the ecological database; (2) choice of the variables that display the best exhaustiveness and reliability; and (3) comparison of these variables in the two databases on the scale of the *SU_analysis* spatial unit.

#### Spatial validation

In spatial terms, the final purpose of the mapping table is to create a background map on the scale of the *SU_analysis* spatial unit. In order to check the quality of the selected spatial unit (*SU_analysis*), it is necessary to evaluate spatial continuity and the decline in spatial resolution.

Spatial continuity is defined as the ability to move from any one point to another point without leaving the spatial unit considered. In other words, a spatially continuous unit has a single boundary [36–38]. A spatial unit that does not meet this condition is referred as discontinuous or fragmented. Most studies of putative links between a health outcome and environmental factors rely on the use of aggregated data. These data are frequently represented by the centroid of each spatial unit. However, in the case of discontinuous spatial units, the centroid may be outside the spatial unit. Hence, an error in the data’s spatial location (due to fragmented spatial units) might affect the findings and result in an erroneous conclusion [36–38]. In order to control for this eventuality, spatial continuity is evaluated by determining the fragmentation of the spatial units, defined as the number of discontinuous *SU_analysis* as a proportion of the total number of *SU_analysis* [37, 38]. This index can be calculated using geographical information systems, such as QGIS and ArcGIS [39, 40].

Spatial resolution is defined as the surface area of the smallest spatial unit in a given data set; it corresponds to the level of detail within the data. Aggregation of spatial units decreases the spatial resolution and thus the quality of the analysis. For example, the spatial resolution decreases if (for a given geographical zone) the data for a town are aggregated with data for the region as a whole. The decline in spatial resolution can initially be evaluated visually. The background map for *SU_analysis* is compared with the background map for the smallest spatial unit in the initial databases, in order to identify any obviously aberrant aggregates. The decline in spatial resolution can then be measured by calculating the ratio between the median surface area of *SU_analysis* and that of the smallest spatial unit in the initial databases (*SU_initial* = *SU_eco* or *SU_med*). This ratio must also be calculated for other administrative reference units whose surface area is known. These ratios are then compared: a lower index of decline corresponds to a spatial unit with a higher spatial resolution.

$$\frac{{{\text{SU\_analysis}}}}{{{\text{SU\_initial}}}}\quad {\text{versus}}\quad \frac{{{\text{SU\_reference1}}}}{{{\text{SU\_initial}}}}\quad {\text{versus}}\quad \frac{{{\text{SU\_reference2}}}}{{{\text{SU\_initial}}}}$$

For example, reference units 1 and 2 could be the county and the state for the USA, or the *canton* and the *département* for France. This index can be also calculated from census data on the number of inhabitants.

## Application of the generic method: an illustrative example based on French databases

### Data sources and objectives

In this section, the generic method is applied to a pair of French medical and ecological databases.

1. The medical database is the PMSI. Collection of these data has been approved by the French National Data Protection Commission (*Commission Nationale de l’Informatique et des Libertés*; authorization 1754053). The database is compiled and released by France’s Technical Agency for Information on Hospitalization (*Agence Technique de l’Information sur l’Hospitalisation*, ATIH). The database contains a summary of each inpatient stay in France, including the ICD-10 diagnostic code, the medical procedures performed (coded according to the French CCAM classification) and the patient’s age, gender, and unique identifier. Each patient is localized by his/her place of residence, which is only characterized by the PMSI spatial ID (*Spatial_Id_PMSI*) in the spatial unit *SU_PMSI*. There were 5644 distinct *SU_PMSI*s in France in 2014, which were characterized by a mean surface area of 97.37 km^2^ and a mean population of 11,174.
2. The ecological database was produced by the INSEE [41]. The INSEE acts as France’s census office, and collects a vast range of demographic, social, economic and housing-related data. Most of the data are publicly available on the INSEE website. The data are summarized for various spatial units: the *commune*, the *canton*, the *département* and the *région* (in increasing hierarchical order; see Additional File 2 for details). Most frequently, the data are summarized on the scale of the *commune* (*SU_INSEE*), which is characterized by the spatial ID *Spatial_Id_INSEE*. In 2014, there were 36,594 *communes* (*SU_INSEE*) in France.
3. The spatial units *SU_PMSI* and *SU_INSEE* differ, as do the IDs *Spatial_Id_PMSI* and *Spatial_Id_INSEE*.

The goal of our method is to create a mapping table for the IDs *Spatial_Id_PMSI* and *Spatial_Id_INSEE*, in order to build a final database that includes both medical data from the PMSI and ecological data from the INSEE. The PMSI medical database provides information on each hospital stay for each patient, which are aggregated for each *Spatial_Id_PMSI* spatial unit. In this illustrative example, the healthcare event of interest is an in-hospital birth. This event was detected by screening for (1) hospital admissions from home, (2) a patient age of 7 days or less, (3) admissions from another hospital with a bodyweight below 2500 g, and (iv) admissions from another hospital, with a patient age below 30 days.

### Construction rules

#### The direction of the relationship

The median [interquartile range (IQR)] surface area is larger for *SU_PMSI* (70 km^2^ [21.6–147.6] than for *SU_INSEE* (10.8 km^2^ [6.4–18.4]. Accordingly, the spatial unit for the analysis (*SU_analysis*) must be based on the spatial unit *SU_PMSI*, which is characterized by the spatial ID *Spatial_Id_PMSI*.

#### Transition matrices M_1_, M_2_

Two transition matrices were required to establish a correlation between *Spatial_Id_INSEE* and *Spatial_Id_PMSI* via the *Zip_code*:
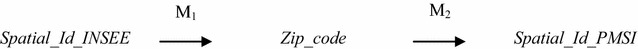


The various equivalence situations for each transition matrix are presented in the Additional file 3. Transition matrix M1 is obtained by correlating *Spatial_Id_INSEE* (the ID for the *communes*) and the *Zip_code* for the *commune* [42]. In France, a zip code corresponds to the geographical zone covered by a single postal delivery office. The equivalence situations are described in detail in Table 1. In over 95% of cases, a given zip code covers several *communes*, which leads to the first data aggregation step (Table 1: situations 1, 4 and 5). In large, highly populated *communes* (< 1%), many zip codes correspond to a single commune. Each zip code corresponds to a single subset of the *commune*, and the union of these distinct subsets constitutes a *commune* (situation 2). In 5% of cases, the zip code corresponds to the *commune*’s *Spatial_Id_INSEE* (situation 3).

**Table 1:**
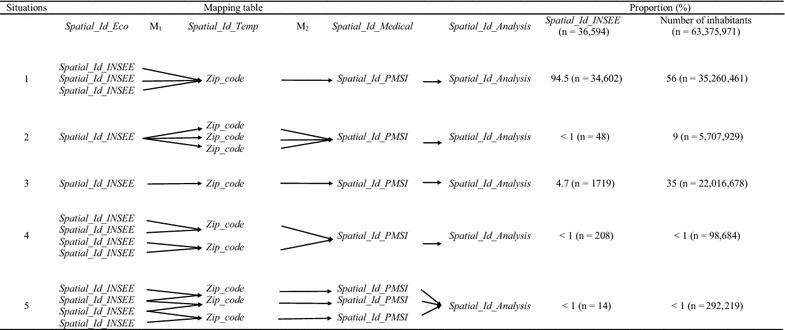
Mapping table for *Spatial_Id_INSEE* and *Spatial_Id_PMSI*

Transition matrix M2 is obtained by correlating *Spatial_Id_PMSI* and *Zip_code*. According to the ATIH, *Spatial_Id_PMSI* has to be built from zip codes for legal reasons [43]. Thus, *Spatial_Id_PMSI* is equivalent to the zip code’s geographic area when the level of statistical confidentiality is high enough (in over 99% of cases; situations 1, 3 and 5). In the opposite case, *Spatial_Id_PMSI* corresponds to the aggregation of several zip codes (< 1% of cases: situations 2 and 4). A second aggregation step is then performed. In situation 2, the transition matrix M1 connects the *commune* to several zip codes. However, data partition is not necessary because the transition matrix M2 aggregates exactly the same units.

Lastly, a *Spatial_Id_analysis* ID is attributed to each of the *Spatial_Id_PMSI* (situations 1 to 4). The combination of transition matrix M1 and transition matrix M2 can, however, generate a small number of particular cases (< 1% of cases). In situation 5, several *Spatial_Id_PMSI* IDs have at least one *Spatial_Id_INSEE* ID in common. It is then impossible to obtain an exact correlation between the spatial ID from the PMSI and the spatial ID from the INSEE. In this situation, the *Spatial_Id_PMSI* IDs are aggregated into a single *Spatial_Id_Analysis* ID. Thus, 23 *Spatial_Id_PMSI* IDs were grouped into 11 *Spatial_Id_Analysis* IDs. In total, there were 5632 *Spatial_Id_Analysis* IDs in the final database.

The data processing and statistical analyses were performed using R software (version 3.3.2) [44]. QGIS software (version 2.14) was used to create the background map and calculate the fragmentation index [39].

### Validation

#### Validation of the mapping table

In order to evaluate the quality of the match between the PMSI database and the INSEE database, the annual number of live births was used as the common variable.

The number of births associated with each *Spatial_Id_INSEE* ID was provided by the INSEE. The number of births associated with each *Spatial_Id_PMSI* ID was obtained by extracting the PMSI database.

For each *Spatial_Id_Analysis* ID, the indicators were compared by calculating the relative difference (i.e. the difference between the number of births in the INSEE data and the number of births in the PMSI data, divided by the number of births in the PMSI data). These relative differences are quoted as the median [IQR]. The total number of births was 785,742 in the INSEE database and 737,545 in the PMSI database, giving a difference of 48,197. The median [IQR] relative difference was 2.3% [0–5.7] (a boxplot is available in the Additional file 4).

In 2012, the ATIH performed an extensive study of the number of inhabitants in each *SU_PMSI* spatial unit, based on the INSEE data. The data on the number of inhabitants are available online for each *Spatial_Id_PMSI* [45]. We therefore transformed these data on the scale of the *Spatial_Id_Analysis* and compared the population data provided by the ATIH and the population data provided for *SU_INSEE*, as aggregated by our mapping table. For each of the *Spatial_Id_Analysis* IDs, the correlation was perfect (difference = 0). Hence, the resulting mapping table automatically performs the task described by the ATIH, regardless of the INSEE variable.

#### Spatial validation

**Fig. 4:**
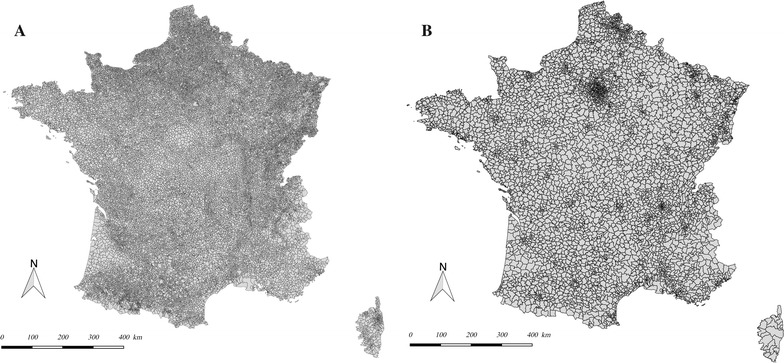
Background map for the *SU_INSEE* (**a**) and *SU_analysis* (**b**) in mainland France in 2014. **a** Background map of the spatial unit *SU_INSEE*, which represents the French *communes* in 2014. **b** Background map of the spatial unit *SU_analysis*, which represents the analysis spatial unit for our application in 2014

A background map of the *SU_analysis* spatial unit was created using data from the French National Geographic Institute (*Institut National de l’Information Géographique et Forestière*, IGN) (Fig. 4). Spatial continuity was evaluated by calculating the fragmentation index; this was 2.4% (n = 134) for the 5632 *SU_analysis* spatial units. This value is within the range of fragmentation indices (2–40%) reported for public use microdata areas (PUMAs) in the USA [37].

A possible decline in the spatial resolution was evaluated first by visual comparison of the respective background maps for *SU_INSEE* and *SU_analysis* (Fig. 4). The *SU_analysis* spatial unit appeared to be regularly distributed over the geographical zone, with no aberrant aggregations. In a second step, we calculated the decline index for the spatial resolution required to obtain data on the surface area and the number of inhabitants in the French *communes*, *cantons* and *départements*. The surface area data came from the IGN, whereas the data on the number of inhabitants came from the INSEE database. The comparison of the spatial resolution index for *SU_analysis* with the French administrative units is described in Table 2. The *SU_analysis* unit has a lower decline index than the *cantons* and *départements* for the surface area (6.5, 13.6 and 555.3, respectively) and the number of inhabitants (14.6, 24 and 1249.2, respectively).

**Table 2:** Comparison of the numbers of inhabitants and surface areas

	N	Surface area^a^			Number of inhabitants^b^		
		Index^c^	Median	IQR^d^	Index^c^	Median	IQR^d^
*SU_reference*							
*Communes*	36,594	*1*	10.8	6.4–18.4	*1*	0.4	0.2–1.1
*Cantons*	3708	*13.6*	146.2	66–209.6	*24*	10.4	5.5–20.4
*Départements*	96	*555.3*	5986	5153–6811	*1249.2*	540.9	306.5–855.8
*SU_analysis*	5632	*6.5*	70	21.6–147.6	*14.6*	6.3	3.5–11.8

Comparison of the numbers of inhabitants and surface areas for French administrative spatial units and the *SU_analysis* spatial unit, via calculation of the decline index for spatial resolution (2014 data)

^a^Surface area (in square kilometres)

^b^Number of inhabitants (in thousands)

^c^Ratio between the median for the spatial unit and the median for the *commune*

^d^Interquartile range

On average, the spatial unit for analysis is therefore 6 times larger than the smallest available unit, testifying to a loss of spatial resolution. However, our method minimizes this loss; for the surface area, the scale is twice as fine as the first reference unit (the *canton*) and nearly 100 times finer than for the *département* (the second reference unit).

As an illustrative example of the application of this method, the birth rate was mapped (Additional file 5).

## Discussion

The method presented here addresses the interoperability problem for ecological and medical databases in a context of the spatial analysis of healthcare events. The loss of spatial resolution was minimized, and we did not have to resort to the use of spatial disaggregation techniques. The method’s application to French national data enabled us to correlate medical data from the PMSI database with ecological data from the INSEE database—resulting in the creation of a final database for fine-scale spatial analysis.

This method may be of value for correlating ecological and medical big data in spatial analyses. This type of data is increasingly available and is opening up new perspectives in epidemiology. However, the use of medical big data in the field of spatial analysis is restrained by interoperability problems, known as the change-of-support problem and the misaligned data problem [26]. In Rossheim et al.’s study of alcohol sales and the socio-economic environment, the data were available on the scale of the zip code, the census block or the zip code tabulation area. To perform analyses on the zip code scale, the researchers were obliged to use spatial disaggregation and aggregation methods; this decreased the quality of the final spatial analysis database [46]. A similar problem was encountered in Sundmacher and Busse’s study of the link between physician supply and avoidable cancer deaths in Germany. The lack of interoperability and the broad range of ecological databases prompted the researchers to use spatial interpolation methods on the district level and to not integrate certain environmental data—thus placing limitations on their analyses [47].

Spatial resolution is a major issue in the spatial analysis of healthcare data because it is easier to detect local phenomena when the resolution is high [30]. For example, the decrease in spatial resolution affects the precision with which a cluster can be localized [8, 48]. The variation in the results of a spatial analysis as a function of the spatial resolution was emphasized by Lee et al.’s study of obesity in the USA; fewer healthcare events were identified when the spatial resolution fell [49]. Jeffery et al. [50] came to a similar conclusion in their study of paediatric leukemia.

The advantage of our method consists in opting for aggregation on the finest scale possible, whilst checking the quality of the final spatial analysis database. This approach appears to have been used previously in a study of stroke, although the method’s details were not specified [23]. The use of spatial disaggregation methods is not desirable, since they lead to a loss of precision in spatial analysis—even when complex models are used [26, 34]. Furthermore, validation of the mapping table results in a high-quality final database for spatial analysis. The spatial validation process ensures that the greatest possible spatial resolution is achieved. Lastly, validation ensured that the spatial units’ fragmentation index remains low. By way of an example, Siordia et al.’s studies of the American PUMA database featured a high fragmentation index and thus encountered theoretical difficulties in the application of statistical models; the spatial position of a healthcare event was no longer coherent with that of a spatial unit [37, 38]. This generic method may provide a structural framework so that researchers can provide a standardized description of the methods used to aggregate ecological and medical spatial data.

Nevertheless, our present method has a number of limitations, most of which are inherent to all spatial analyses. Firstly, a large percentage of the scenario 5 (Table 1) might decrease the spatial resolution, due to the aggregation of several basic spatial units. This issue can be evaluated by analyzing the spatial resolution index (as presented in the present study) and establishing whether the final spatial unit sizes are homogeneously distributed or not. Secondly, the geographical boundaries of spatial units change over time, which can make it more difficult to study healthcare events over a long time interval. This problem can be tackled in two ways: by optimizing the study period and thus minimizing changes in geographical boundaries or by considering the geographic boundaries that correspond to the longest study period. Thirdly, our method only partly addressed the change-of-support problem because it only applies to aggregated data (a frequent situation in the spatial analysis of healthcare events, nevertheless) [51]. Therefore, for other types of spatial data (such as geostatistical data), preliminary work on aggregation to the spatial unit of interest must be carried out in collaboration with specialists in the particular field. Lastly, the present method requires the definition of transition matrices prior to construction of the mapping table.

## Conclusion

In conclusion, the present work suggests that it is possible to significantly improve the interoperability of ecological databases and medical databases, and thus enable finer-scale analyses. In view of the growing availability of big data, the method presented here could be a useful tool for the precise spatial analysis of large geographical areas.

## Additional files

**Additional file 1**. Illustrative example of the method for building a final database for spatial analysis.

**Additional file 2**. Description of the French administrative spatial units in terms of frequencies, surface area (in km^2^) and number of inhabitants. The different circles indicate the hierarchical relationships between the different administrative units.

**Additional file 3**. Equivalence situations for the transition matrices M_1_ and M_2_. Matrix 1 is the tool used to link the Spatial_Id_INSEE and the Zip_Code. Matrix 2 is the tool used to link the Zip_Code and the Spatial_Id_PMSI. “Yes” indicates situations encountered in the application.

**Additional file 4**. Relative difference in the number of births per spatial unit *SU_analysis* between the ecological data from the INSEE and the medical data from the PMSI.

**Additional file 5**. Birth rate for 10,000 inhabitants for the *SU_INSEE* (A) and *SU_analysis* (B). The first map (A) is on the scale of the *commune* spatial unit (*SU_INSEE*), and represents the birth rate calculated from the number of births and the underlying population data in the INSEE database. The second map (B) is on the scale of the *SU_analysis* spatial unit, and represents the birth rate per 10,000 inhabitants calculated using the number of births in the PMSI database and the underlying population data in the INSEE database.

## Abbreviations

PMSI: Programme de Médicalisation des Systèmes d’Information
INSEE: Institut National de la Statistique et des Etudes Economiques
ATIH: Agence Technique de l’Information sur l’Hospitalisation
IQR: interquartile range
IGN: Institut National de l’Information Géographique et Forestière
PUMA: public use microdata area
