# Supplementary material for: A generic method for improving the spatial interoperability of medical and ecological databases
Source: Int J Health Geogr. 2017 Oct 3;16:36. doi: 10.1186/s12942-017-0109-5 (PMC5627422; doi:10.1186/s12942-017-0109-5)
Supplement: Supplementary file 1 — Additional file 1. Illustrative example of the method for building a final database for spatial analysis. [file 12942_2017_109_MOESM1_ESM.pdf]

Ecological database

Spatial_Id_Eco	Number of inhabitants	% of unemployment
North_West	2587	8.6
South_East	965	6.9

Medical database

ID patient	***Spatial_Id_medical***	Diabetes
1	North	1
2	South	0
3	West	1
4	North	0
5	West	1
6	East	0

Aggregation

Spatial_Id_medical	Number of diabete cases
North	1
East	0
South	0
West	2

Mapping table

Spatial_Id_Eco	***Spatial_Id_medical***
North_West	North
North_West	West
South_East	South
South_East	East

Final spatial analysis database

Spatial_Id_analyse	Number of inhabitants	% of unemployment	Number of diabete cases
North_West	2587	8.6	3
South_East	965	6.9	0
