# Supplementary material for: A generic method for improving the spatial interoperability of medical and ecological databases
Source: Int J Health Geogr. 2017 Oct 3;16:36. doi: 10.1186/s12942-017-0109-5 (PMC5627422; doi:10.1186/s12942-017-0109-5)
Supplement: Supplementary file 2 — Additional file 2. Description of the French administrative spatial units in terms of frequencies, surface area (in km2) and number of inhabitants. The different circles indicate the hierarchical relationships between the different administrative units. [file 12942_2017_109_MOESM2_ESM.pdf]

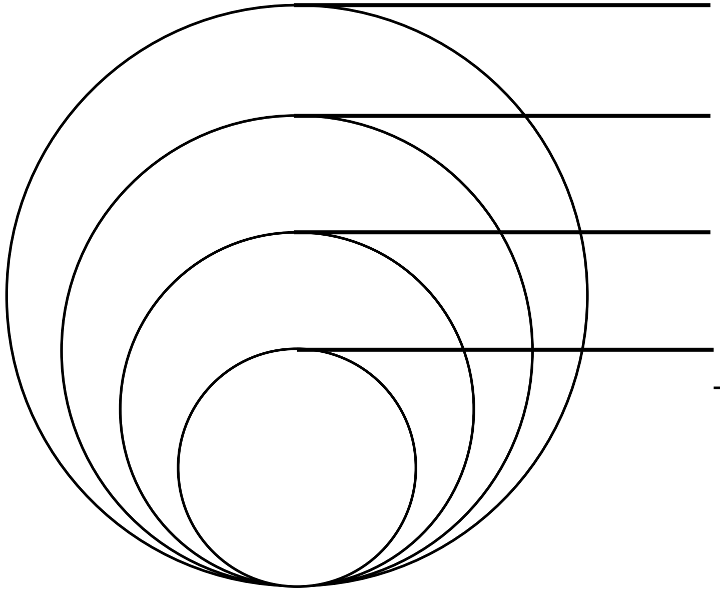

Administrative area	Count	Median surface area (km^2)	Median number of inhabitants
Régions	18	23,669	2,136,100
Départements	96	5,987	540,900
Cantons	4,055	146	10,400
Communes	36,594	11	400
