# Supplementary material for: A generic method for improving the spatial interoperability of medical and ecological databases
Source: Int J Health Geogr. 2017 Oct 3;16:36. doi: 10.1186/s12942-017-0109-5 (PMC5627422; doi:10.1186/s12942-017-0109-5)
Supplement: Supplementary file 3 — Additional file 3. Equivalence situations for the transition matrices M1 and M2. Matrix 1 is the tool used to link the Spatial_Id_INSEE and the Zip_Code. Matrix 2 is the tool used to link the Zip_Code and the Spatial_Id_PMSI. “Yes” indicates situations encountered in the application. [file 12942_2017_109_MOESM3_ESM.pdf]

Situations	Matrix 1	Matrix 2
	Spatial_Id_INSEE ⟷ *Zip_Code*	*Zip_Code* ⟷ *Spatial_Id_PMSI*
**1** ⟷ 1	Yes	Yes
**n** ⟷ 1	Yes	Yes
**1** ⟷ n	Yes	No
**n** ⟷ n	No	No
