# Supplementary figures and images for: A generic method for improving the spatial interoperability of medical and ecological databases
Source: Int J Health Geogr. 2017 Oct 3;16:36. doi: 10.1186/s12942-017-0109-5 (PMC5627422; doi:10.1186/s12942-017-0109-5)

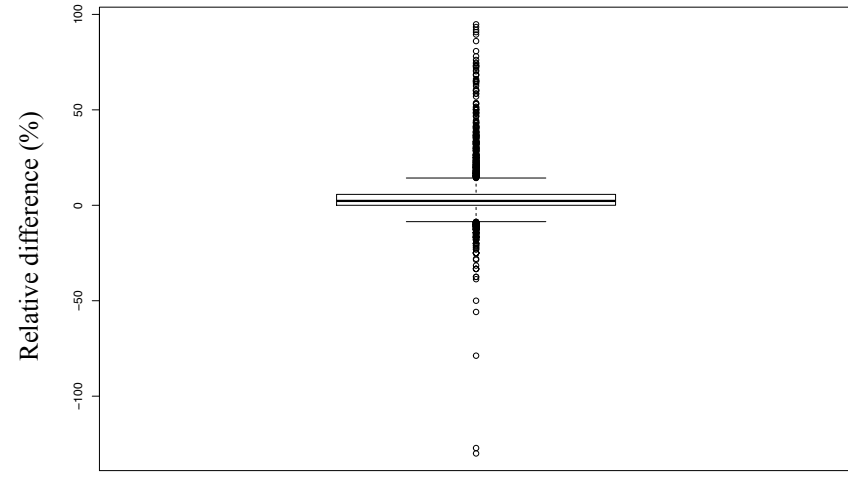

Supplement: Supplementary file 4 — Additional file 4. Relative difference in the number of births per spatial unit SU_analysis between the ecological data from the INSEE and the medical data from the PMSI. [file 12942_2017_109_MOESM4_ESM.pdf]

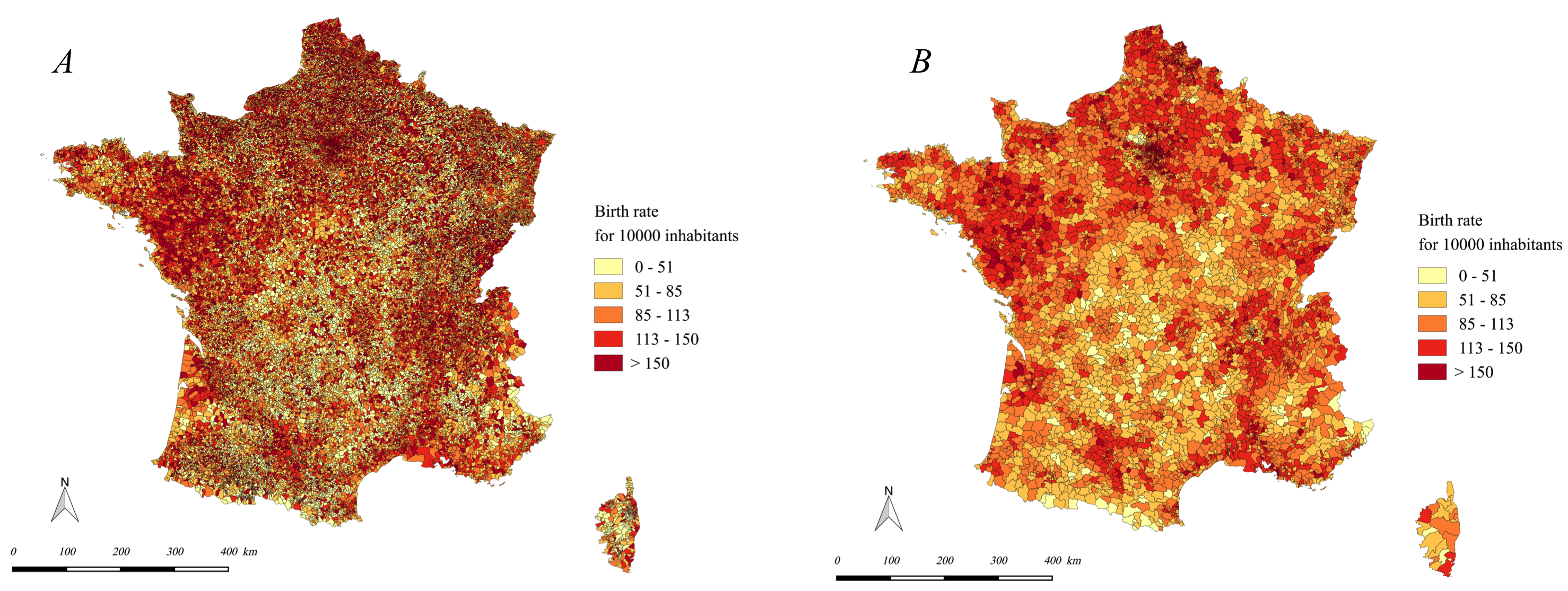

Supplement: Supplementary file 5 — Additional file 5. Birth rate for 10,000 inhabitants for the SU_INSEE (A) and SU_analysis (B). The first map (A) is on the scale of the commune spatial unit (SU_INSEE), and represents the birth rate calculated from the number of births and the underlying population data in the INSEE database. The second map (B) is on the scale of the SU_analysis spatial unit, and represents the birth rate per 10,000 inhabitants calculated using the number of births in the PMSI database and the underlying population data in the INSEE database. [file 12942_2017_109_MOESM5_ESM.png]
